# A Novel Alpha-Power X Family: A Flexible Framework for Distribution Generation with Focus on the Half-Logistic Model

**DOI:** 10.3390/e27060632

**Published:** 2025-06-13

**Authors:** A. A. Bhat, Aadil Ahmad Mir, S. P. Ahmad, Badr S. Alnssyan, Abdelaziz Alsubie, Yashpal Singh Raghav

**Affiliations:** 1 Department of Mathematical Sciences, Islamic University of Science and Technology, Awantipora 192122, India; 2 Department of Statistics, University of Kashmir, Srinagar 190006, India; aadilmir.stscholar@kashmiruniversity.net (A.A.M.); sprvz@uok.edu.in (S.P.A.); 3 Department of Management Information Systems, College of Business and Economics, Qassim University, Buraydah 51452, Saudi Arabia; 4 Department of Basic Sciences, College of Science and Theoretical Studies, Saudi Electronic University, Riyadh 11673, Saudi Arabia; a.alsubie@seu.edu.sa; 5 Department of Mathematics, College of Science, Jazan University, P.O. Box 2097, Jazan 45142, Saudi Arabia; yraghav@jazanu.edu.sa

**Keywords:** novel alpha-power X family, statistical model, statistical characterization, half-logistic distribution, estimation approaches, simulation, real-world applications

## Abstract

This study introduces a new and flexible class of probability distributions known as the novel alpha-power X (NAP-X) family. A key development within this framework is the novel alpha-power half-logistic (NAP-HL) distribution, which extends the classical half-logistic model through an alpha-power transformation, allowing for greater adaptability to various data shapes. The paper explores several theoretical aspects of the proposed model, including its moments, quantile function and hazard rate. To assess the effectiveness of parameter estimation, a detailed simulation study is conducted using seven estimation techniques: Maximum likelihood estimation (MLE), Cramér–von Mises estimation (CVME), maximum product of spacings estimation (MPSE), least squares estimation (LSE), weighted least squares estimation (WLSE), Anderson–Darling estimation (ADE) and a right-tailed version of Anderson–Darling estimation (RTADE). The results offer comparative insights into the performance of each method across different sample sizes. The practical value of the NAP-HL distribution is demonstrated using two real datasets from the metrology and engineering domains. In both cases, the proposed model provides a better fit than the traditional half-logistic and related distributions, as shown by lower values of standard model selection criteria. Graphical tools such as fitted density curves, Q–Q and P–P plots, survival functions and box plots further support the suitability of the model for real-world data analysis.

## 1. Introduction

In the field of statistical distribution theory, the practice of introducing additional parameters to existing distribution families has become widespread and highly valuable. By adding an extra parameter, statisticians are able to significantly enhance the flexibility of the underlying models, allowing them to better capture complex data patterns and provide improved fits to a variety of real-world phenomena. This approach not only enriches the mathematical structure of distributions but also extends their practical applications across numerous scientific and engineering disciplines.

Since the 1980s, the development of new probability distributions has largely focused on two principal strategies: one is the combination of existing distributions to create entirely new families and the other is the extension of classical distributions by introducing one or more extra parameters. The former method offers a way to blend the strengths of different models, while the latter provides a systematic enhancement of a given model’s flexibility without altering its core characteristics.

Adding new parameters often allows distributions to adapt to a broader range of shapes, including skewed, heavy-tailed or multi-modal data structures, which are frequently encountered in practice. Such improvements make the extended models particularly useful for applications in fields like survival analysis, reliability engineering, finance, environmental studies and biomedical research. Moreover, these extended families maintain a close connection to the baseline distributions, ensuring that the original models remain special or limiting cases within the new, more general, framework.

Overall, the introduction of additional parameters into existing distribution families continues to be a powerful and essential technique in statistical modeling. It not only broadens the theoretical landscape of probability distributions but also significantly enhances their practical utility in analyzing and interpreting real-world data.

One of the earliest and most fundamental techniques for generating new probability distributions was introduced in Reference [1], which proposed the exponentiated family of distributions. This approach enhances a baseline distribution by introducing an additional shape parameter, thereby increasing its flexibility to model a wider variety of data patterns. It is widely acknowledged that no single probability model consistently provides the best fit across all real-world scenarios. As a result, various fields in statistics continually seek to develop new probability models tailored to specific needs. Modified or newly developed distributions often offer better fitting capabilities compared to existing models, which motivates researchers to explore novel distributions applicable across diverse areas. The cumulative distribution function (CDF) of the exponentiated family is given by

(1) $$F\left(x;\gamma ,\zeta \right)={\left[G(x;\zeta )\right]}^{\gamma },\;\gamma ,\zeta >0,\;x\in R.$$

Here, γ>0 serves as an additional shape parameter and G(x;ζ) represents the CDF of the baseline random variable with parameter vector ζ. The inclusion of this extra parameter enhances the classical distributions’ ability to better capture complex real-world phenomena.

Building on this idea, Reference [2] introduced the Marshall–Olkin family of distributions, aimed at further improving the flexibility of existing models. Their transformation modifies the baseline CDF using the parameter α, resulting in the following CDF:

(2) $${\bar{F}}_{MO}\left(\alpha ;x\right)=\frac{\alpha \bar{F}\left(x\right)}{1-\bar{\alpha }\bar{F}\left(x\right)},\;\alpha >0,\;\bar{\alpha }=1-\alpha ,\;x\in R.$$

This transformation offers better control over the tail behavior of distributions, making it particularly useful in reliability and risk analysis.

To introduce greater flexibility in probability modeling, Reference [3] proposed the alpha-power transformation (APT), which incorporates a new parameter α into the baseline distribution G(x;β). The properties of the exponential distribution under this transformation were discussed as a special case. The CDF of the APT family is given by

(3) $$F\left(x;\alpha ,\zeta \right)=\left\{\begin{array}{cc} \frac{\alpha^{G(x;\zeta )}-1}{\alpha -1}, & \mathrm{if}\;\alpha >0,\;\alpha \neq 1,\\ G(x;\zeta ), & \mathrm{if}\;\alpha =1. \end{array}\right.$$

Further enhancing flexibility, Reference [4] introduced the MIT transformation, which modifies a given baseline CDF by introducing a shape parameter α. Under the MIT framework, the transformed CDF is expressed as

(4) $$F_{MIT}\left(x;\alpha \right)=\frac{\alpha F(x)}{1-\bar{\alpha }F\left(x\right)},\;\alpha >0,\;\bar{\alpha }=1-\alpha ,\;x\in R.$$

Additionally, Reference [5] applying the T-X family methodology from [6], proposed the new exponent power-X (NGEP-X) family of distributions to generate more flexible probability models. The cumulative distribution function (CDF) of the NGEP-X family is given by

(5) $$F\left(x;\alpha ,\zeta \right)=1-\left(e^{\alpha G(x;\zeta )}-e^{\alpha }G\left(x;\zeta \right)\right),\;\alpha >0,\;x\in R.$$

In recent developments, researchers have increasingly explored trigonometric transformations in constructing new distributions. For instance, Reference [7] introduced a trigonometric function-based family of distributions, where a trigonometric function inside the exponent transforms the baseline distribution. The corresponding CDF is given by

(6) $$F\left(x;\zeta \right)=\frac{e^{1-\cos\left(\frac{\pi G(x;\zeta )}{1+G(x;\zeta )}\right)}-1}{e-1},\;\zeta >0,\;x\in R.$$

Continuing in this direction, Reference [8] introduced a flexible family of distributions designed to better model skewed and heavy-tailed data, offering robust alternatives to classical models, especially in engineering and medical sciences. The corresponding CDF is given by

(7) $$F_{\mathrm{ASP}}\left(x;\alpha ,\zeta \right)=2\sin\left(\frac{\pi }{2}{\left[C(x;\zeta )\right]}^{\alpha }\right)-{\left[C(x;\zeta )\right]}^{\alpha },\;\alpha ,\zeta >0,\;x\in R.$$

Moreover, Reference [9] introduced the Zubair-G family of distributions, with the CDF defined as

(8) $$F\left(x;\alpha ,\xi \right)=\frac{e^{\alpha G{\left(x;\xi \right)}^{2}}-1}{e^{\alpha }-1},\;\alpha ,\xi >0,\;x\in R.$$

Similarly, Reference [10] proposed the modified alpha-power exponential (MAPE) distribution, with the CDF given by

(9) $$G\left(x\right)=\frac{\beta^{F^{2}\left(x\right)}\alpha^{F(x)}-1}{\alpha \beta -1},\;\alpha ,\beta \geq 1,\;\alpha \beta \neq 1.$$

The extended alpha-power-transformed (Ex-APT) family, introduced in [11], has its CDF expressed as

(10) $$G\left(x;\alpha ,\xi \right)=\frac{\alpha^{F(x;\xi )}-e^{F(x;\xi )}}{\alpha -e},\;\xi >0,\;\alpha \neq e,\;\alpha >e,\;x\in R.$$

Finally, Reference [12] proposed the alpha–beta power-E (ABPE) transformation method, with the CDF given by

(11) $$G\left(z\right)=\frac{\alpha^{F(z)}-\beta^{F(z)}}{\alpha -\beta },\;z\in R,\;\alpha ,\beta >0,\;\alpha \neq \beta \neq 1.$$

Extending recent developments in probability distribution generation, this study proposes the novel and versatile novel alpha-power X (NAP-X) family. Traditional enhancement techniques often introduce additional shape parameters, potentially leading to parameter redundancy, increased complexity and difficulties in interpretation and implementation. The NAP-X framework strategically overcomes these limitations by providing enhanced distributional flexibility without unnecessary parameter inflation.

The NAP-X family is a transformation-based framework that systematically modifies baseline distributions to achieve superior adaptability. Unlike conventional methods relying on direct parameter expansion, it employs a controlled transformation mechanism to adjust shape and tail behavior. This enables the efficient modeling of both symmetric and asymmetric data patterns, making it a powerful tool for complex real-world phenomena across diverse fields.

As a specific illustration, we derive a modified half-logistic distribution via the NAP-X transformation. This model demonstrates enhanced performance, particularly in its probability density function (PDF) and hazard rate function (HRF). Comprehensive comparisons with established models—including the alpha-power exponential and Marshall–Olkin exponential distributions—using standard metrics (AIC, BIC, AICC, HQIC, K-S statistics) consistently show the superior flexibility and goodness of fit of the NAP-X model, detailed in the applications sections.

Overall, the NAP-X family preserves the theoretical elegance of classical distributions while offering a robust mechanism for capturing diverse data patterns. Its effective handling of symmetry and asymmetry renders it highly suitable for reliability analysis, survival studies, biomedical research and engineering systems.

The manuscript is structured as follows:

- Section 2: Presents the motivation behind this study and highlights the research gap addressed by the proposed NAP-X family of distributions.
- Section 3: Introduces the novel alpha-power X (NAP-X) family of distributions and its special case, the NAP-HL model, along with key mathematical properties.
- Section 4: Discusses parameter estimation using seven classical estimation techniques.
- Section 5: Conducts a comprehensive Monte Carlo simulation study to evaluate and compare the performance of the proposed estimators across varying sample sizes.
- Section 6: Validates the practical applicability of the NAP-HL model through real-world datasets.
- Section 7: Concludes the manuscript with major contributions, findings and directions for future research.

A defining feature of the NAP-X family is its introduction of a shape parameter α>0 to generalize baseline distributions. This parameter enables the modeling of diverse behaviors such as skewness, varying tail weights and flexible hazard rate shapes, often inadequately captured by standard models. Crucially, the baseline distribution is retained as the special case when α=1, ensuring the NAP-X family is a true generalization that preserves baseline properties while offering enhanced flexibility. This makes it particularly valuable for survival analysis, reliability studies and industrial data modeling.

## 2. Motivation and Research Gap

The advancement of statistical modeling, particularly in reliability and survival analysis, has led to the creation of numerous flexible probability distributions. These models aim to better capture real-world data behavior, especially in terms of skewness, kurtosis and hazard rate dynamics. A popular method to achieve flexibility involves transforming existing baseline distributions using functional techniques such as the exponentiated, Marshall–Olkin, alpha-power, beta-generated and transmutation approaches.

While these approaches have yielded substantial improvements in model fitting, they often come at the cost of excessive parameterization. The addition of multiple parameters can lead to interpretational difficulties, overfitting, numerical instability in estimation and challenges in model selection. Furthermore, many classical transformations lack the ability to simultaneously control symmetry and tail behavior in a simple yet effective manner.

In particular, the alpha-power transformation, despite its wide applicability, has limited structural flexibility when applied directly. It modifies tail behavior but does not consistently enhance hazard rate shapes or allow symmetric modeling. Moreover, transformations such as the Marshall–Olkin method tend to create abrupt changes in distribution structure, which may not be suitable for gradual changes observed in empirical data.

To address these limitations, there is a need for a new transformation technique with the following properties:

- Enhances baseline distributions while maintaining parsimony (i.e., minimal parameter addition);
- Improves flexibility in modeling both symmetric and asymmetric data;
- Provides a wide variety of hazard rate shapes;
- Is adaptable to various fields such as bio-statistics, engineering and economics.

This motivates the development of the novel alpha-power X (NAP-X) family of distributions, which generalizes the alpha-power transformation in a more structurally sound and application friendly manner. Unlike conventional methods, the NAP-X transformation introduces a shape control mechanism that modifies the baseline distribution with minimal structural disruption and parameter inflation. The resulting family offers significant improvements in terms of fitting ability, interpretability and applicability to real-life datasets.

## 3. Novel Alpha-Power X Family and Properties

In this section, we introduce a novel and flexible class of probability distributions, termed the novel alpha-power (NAP) X family. The primary objective behind the formulation of this family is to enhance the adaptability of existing probabilistic models by integrating an additional shape parameter. This newly introduced parameter enables simultaneous control over the shape and scale features of a given baseline distribution, thereby offering improved modeling capabilities for complex real-world data encountered in diverse domains such as engineering, finance and survival analysis.

The NAP-X family is generated by applying a transformation to the cumulative distribution function (CDF) of a baseline distribution, denoted by H(x). This transformation incorporates a shape parameter α>0, which plays a crucial role in regulating the tail behavior and improving the overall flexibility of the resulting distribution. Mathematically, the CDF of the NAP-X family is expressed as follows.

**Definition 1.**  *Let H(x) represent the CDF of the reference model. Then, the CDF F(x) of the NAP-X family distribution is obtained as*

(12) $$F\left(x\right)=\frac{\alpha^{1-{\left[H\left(x\right)\right]}^{\alpha }}-\alpha }{1-\alpha };\;x>0,\;\alpha >0,\;\alpha \neq 1$$

In order to determine whether Equation (12) is accurate, we must prove the following lemma.

**Lemma 1.**  *The CDF F(x) obtained in Equation (12) is exact if *

$$\lim_{x\to -\infty }F\left(x\right)=0\;\mathrm{and}\lim_{x\to \infty }F\left(x\right)=1.$$

**Proof.**  We begin by considering the following:

$$\lim_{x\to -\infty }F\left(x\right)=\frac{\alpha^{1-{\left[H\left(x\right)\right]}^{\alpha }}-\alpha }{1-\alpha },$$

$$\Rightarrow \lim_{x\to -\infty }F\left(x\right)=\frac{\alpha^{1-{\left[H\left(-\infty \right)\right]}^{\alpha }}-\alpha }{1-\alpha }=0.$$

Also,

$$\begin{array}{cc} \lim_{x\to \infty }F\left(x\right) & =\frac{\alpha^{1-{\left[H\left(x\right)\right]}^{\alpha }}-\alpha }{1-\alpha },\\ & =\frac{\alpha^{1-{\left[H\left(\infty \right)\right]}^{\alpha }}-\alpha }{1-\alpha }=1. \end{array}$$

   □

**Property 1.**  *The CDF F(x;γ,ζ) derived in Equation (12) is right-continuous and differentiable.*

$$\frac{d}{dx}F\left(x;\gamma ,\zeta \right)=f\left(x;\gamma ,\zeta \right).$$

*Thus, based on lemma and property, we have determined that the CDF given in Equation (12) is valid. The corresponding PDF of the AP-X family, derived from Equation (12), is provided as*

(13) $$f\left(x\right)=\frac{\alpha \log\alpha {\left[H\left(x\right)\right]}^{\alpha -1}h\left(x\right)}{\alpha -1}\alpha^{1-{\left[H\left(x\right)\right]}^{\alpha }},\;x>0,\;\alpha >0,\;\alpha \neq 1$$

*Here, $h\left(x\right)=\frac{d}{dx}H\left(x\right)$ is the PDF of the reference model and H(x) is the baseline CDF.*

- When α=1, Equation (12) reduces to H(x), the cumulative distribution function (CDF) of the baseline distribution.

### 3.1. Novel Alpha-Power Half-Logistic Model

In this subsection of the article, a particular subclass of the NAP-X family, characterized by two parameters, is introduced. We refer to this particular model as the novel alpha-power half-logistic (NAP-HL) model.

The CDF H(x) and PDF h(x) of the half-logistic distribution are defined as follows:

(14) $$H\left(x\right)=\frac{1-e^{-\theta x}}{1+e^{-\theta x}},\;\mathrm{so}\;1-H\left(x\right)=\frac{2e^{-\theta x}}{1+e^{-\theta x}}$$

(15) $$h\left(x\right)=\frac{2\theta e^{-\theta x}}{{\left(1+e^{-\theta x}\right)}^{2}}$$

By inserting Equations (14) and (15) into Equations (12) and (13), we obtain the updated version of the NAP half-logistic (NAP-HL) model, as expressed by

(16) $$F\left(x\right)=\frac{\alpha^{1-{\left(\frac{1-e^{-\theta x}}{1+e^{-\theta x}}\right)}^{\alpha }}-\alpha }{1-\alpha },\;x>0,\;\alpha >0,\;\alpha \neq 1.$$

and the PDF takes the form

(17) $$f\left(x\right)=\frac{\alpha \log\alpha }{\alpha -1}{\left(\frac{1-e^{-\theta x}}{1+e^{-\theta x}}\right)}^{\alpha -1}\frac{2\theta e^{-\theta x}}{{\left(1+e^{-\theta x}\right)}^{2}}\;\alpha^{1-{\left(\frac{1-e^{-\theta x}}{1+e^{-\theta x}}\right)}^{\alpha }},\;x>0,\;\alpha >0,\;\alpha \neq 1.$$

The statistical properties including Survival Function S(x), Hazard Rate Function h(x), Reverse Hazard Function m(x) and Cumulative Hazard Function H(x) for the special sub-model are derived as follows:

(18) $$S\left(x\right)=\frac{1-\alpha^{1-{\left(\frac{1-e^{-\theta x}}{1+e^{-\theta x}}\right)}^{\alpha }}}{1-\alpha }.$$

(19) $$h\left(x\right)=\frac{\alpha \log\alpha }{\alpha^{1-{\left(\frac{1-e^{-\theta x}}{1+e^{-\theta x}}\right)}^{\alpha }}-1}{\left(\frac{1-e^{-\theta x}}{1+e^{-\theta x}}\right)}^{\alpha -1}\frac{2\theta e^{-\theta x}}{{\left(1+e^{-\theta x}\right)}^{2}}\;\alpha^{1-{\left(\frac{1-e^{-\theta x}}{1+e^{-\theta x}}\right)}^{\alpha }}.$$

**Figure 1:**
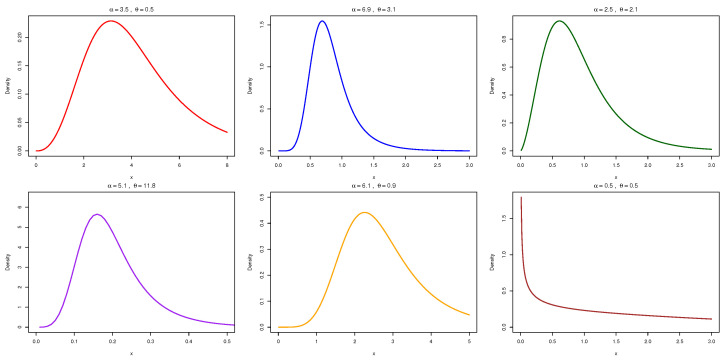
PDF curves of the NAP-HL model for different parameter settings of α and θ.

**Figure 2:**
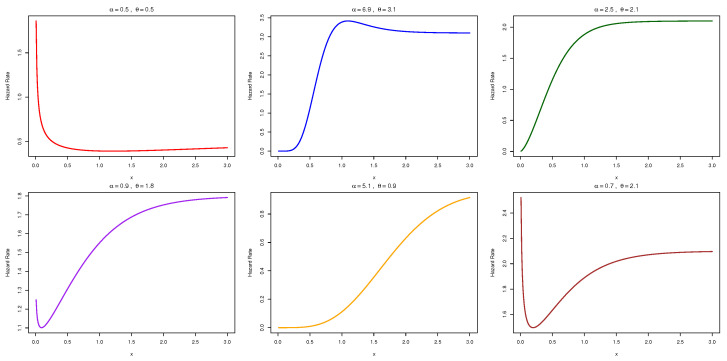
Diverse hazard rate shapes of the NAP-HL model for different settings of α and θ.

Figure 1 and Figure 2 illustrates the behavior of the PDF and HRF for the NAP-HL model under varying parameter conditions.

(20) $$m\left(x\right)=\frac{\alpha \log\alpha }{\alpha -\alpha^{1-{\left(\frac{1-e^{-\theta x}}{1+e^{-\theta x}}\right)}^{\alpha }}}{\left(\frac{1-e^{-\theta x}}{1+e^{-\theta x}}\right)}^{\alpha -1}\frac{2\theta e^{-\theta x}}{{\left(1+e^{-\theta x}\right)}^{2}}\;\alpha^{1-{\left(\frac{1-e^{-\theta x}}{1+e^{-\theta x}}\right)}^{\alpha }}.$$

and

(21) $$H\left(x\right)=\log\left\{\frac{1-\alpha }{1-\alpha^{1-{\left(\frac{1-e^{-\theta x}}{1+e^{-\theta x}}\right)}^{\alpha }}}\right\}$$

In addition, the QF of *X* for the following NAP-HL model is expressed as

(22) $$X_{u}=-\frac{1}{\theta }\log\left\{\frac{\log\left[\frac{1}{u(1-\alpha )+\alpha }\right]}{\log\left(\frac{\alpha^{2}}{u(1-\alpha )+\alpha }\right)}\right\}\;;\;0<u<1.$$

#### 3.1.1. Expansion Form

The PDF of the NAP-HL model can be written as

(23) $$f\left(x\right)=\frac{\alpha^{2}\log\alpha }{\alpha -1}{\left(\frac{1-e^{-\theta x}}{1+e^{-\theta x}}\right)}^{\alpha -1}\frac{2\theta e^{-\theta x}}{{\left(1+e^{-\theta x}\right)}^{2}}\;\alpha^{-{\left(\frac{1-e^{-\theta x}}{1+e^{-\theta x}}\right)}^{\alpha }}$$

We begin by using the exponential expansion of $\alpha^{x}$, which is

(24) $$\alpha^{-x}=\sum_{k=0}^{\infty }\frac{{\left(-1\right)}^{k}{\left(\log\alpha \right)}^{k}x^{k}}{k!};\;\left|x\right|<1.$$

Thus, the PDF becomes

(25) $$\begin{array}{cc} f(x) & =\frac{2\alpha^{2}\theta \log\alpha }{\alpha -1}\sum_{k=0}^{\infty }\frac{{\left(-1\right)}^{k}{\left(\log\alpha \right)}^{k}}{k!}\frac{{\left(1-e^{-\theta x}\right)}^{\alpha (k+1)-1}}{{\left(1+e^{-\theta x}\right)}^{\alpha (k+1)+1}}e^{-\theta x}. \end{array}$$

Using the binomial expansion

(1−x)r=∑p=0∞rp(−1)pxp,;|x|<1

(1+x)−s=∑q=0∞(−1)qs+q−1qxq;|x|<1.

we get

(26) f(x)=2α2θα−1∑k=0∞∑p=0∞∑q=0∞(−1)k+p+q(logα)k+1k!α(k+1)−1pα(k+1)+kqe−θ(p+q+1)x.

Combining constants into a single coefficient, we define

(27) δk,p,q=(−1)k+p+q(logα)k+1k!α(k+1)−1pα(k+1)+kq.

Thus, the PDF of the NAP-HL model can be written in the mixture representation, respectively, as

(28) $$f\left(x\right)=\frac{2\alpha^{2}\theta }{\alpha -1}\sum_{k,p,q=0}^{\infty }\delta_{k,p,q}\;e^{-\theta (p+q+1)x}.$$

#### 3.1.2. Moments and Moment Generating Function

For the NAP-HL model, the ordinary *r*th moment is calculated as

(29) $${\mu_{r}}^{\prime }=\int_{0}^{\infty }x^{r}f\left(x\right)dx.$$

Substituting Equation (28) into Equation (29), we get

(30) $${\mu_{r}}^{\prime }=\frac{2\alpha^{2}\theta }{\alpha -1}\sum_{k,p,q=0}^{\infty }\delta_{k,p,q}\frac{\Gamma (r+1)}{{\left(\left(p+q+1\right)\theta \right)}^{r+1}}$$

Putting r=1 into Equation (36), the mean of the NAP-HL model is computed as

(31) $${\mu_{1}}^{\prime }=\frac{2\alpha^{2}\theta }{\alpha -1}\sum_{k,p,q=0}^{\infty }\delta_{k,p,q}\frac{1}{{\left(\left(p+q+1\right)\theta \right)}^{2}}$$

Using the series representation of $e^{tx}$, we have

(32) $$M_{X}\left(t\right)=\sum_{r=0}^{\infty }\frac{t^{r}}{r!}E\left(X^{r}\right)$$

Substituting the value of Equation (30) into Equation (32), we get the moment generating function of the NAP-HL model as

(33) $$M_{X}\left(t\right)=\frac{2\alpha^{2}\theta }{\alpha -1}\sum_{k,p,q,r=0}^{\infty }\delta_{k,p,q}\;\frac{t^{r}}{r!}\frac{\Gamma (r+1)}{{\left(\left(p+q+1\right)\theta \right)}^{r+1}}$$

Table 1 presents a summary of the moments, mode, variance and shape measures (skewness and kurtosis) for selected combinations of (α,θ) parameter values, demonstrating the sensitivity of the distribution to parameter changes.

#### 3.1.3. *r*th Incomplete Moment

The *r*th incomplete moment is defined as

(34) $$\phi_{r}\left(z\right)=\int_{0}^{z}x^{r}f\left(x\right)dx$$

Substituting Equation (28) into Equation (34), then the *r*th incomplete moment of NAP-HL model is given by

(35) $$\phi_{r}\left(z\right)=\frac{2\alpha^{2}\theta }{\alpha -1}\sum_{k,p,q=0}^{\infty }\delta_{k,p,q}\frac{1}{{\left(\left(p+q+1\right)\theta \right)}^{r+1}}\gamma \left(\left(p+q+1\right)\theta \right),r+1).$$

Putting r=1 into Equation (35), we get the first incomplete moment as

(36) $$\phi_{1}\left(z\right)=\frac{2\alpha^{2}\theta }{\alpha -1}\sum_{k,p,q=0}^{\infty }\delta_{k,p,q}\frac{1}{{\left(\left(p+q+1\right)\theta \right)}^{2}}\gamma \left(\left(p+q+1\right)\theta \right),2).$$

#### 3.1.4. Mean Residual Life (MRL) and Mean Waiting Time (MWT)

The MRL for the NAP-HL model is given by

$$\mu \left(t\right)=\frac{1-\alpha }{1-\alpha^{1-{\left(\frac{1-e^{-\theta x}}{1+e^{-\theta x}}\right)}^{\alpha }}}\left[\frac{2\alpha^{2}\theta }{\alpha -1}\sum_{k,p,q=0}^{\infty }\delta_{k,p,q}\frac{1}{{\left(\left(p+q+1\right)\theta \right)}^{2}}\left(1-\gamma ((p+q+1)\theta ),2)\right)\right]-t$$

The MWT for the NAP-HL model is given by

(37) $$\bar{\mu }\left(t\right)=t-\frac{2\alpha^{2}\theta \sum_{k,p,q=0}^{\infty }\delta_{k,p,q}\frac{1}{{\left(\left(p+q+1\right)\theta \right)}^{2}}\gamma \left(\left(p+q+1\right)\theta \right),2)}{\alpha -\alpha^{1-{\left(\frac{1-e^{-\theta x}}{1+e^{-\theta x}}\right)}^{\alpha }}}$$

#### 3.1.5. Rényi Entropy

The Rényi entropy of order ϕ for the NAP-HL model is defined as

(38) $$R_{\phi }=\frac{1}{1-\phi }\log\left[\int_{0}^{\infty }f{\left(x\right)}^{\phi }\;dx\right],\;\phi >0,\;\phi \neq 1,\;x\in R.$$

By substituting the expression of f(x) from Equation (28) into Equation (38), the Rényi entropy of order ϕ for the NAP-HL model becomes

(39) $$R_{\phi }=\frac{1}{1-\phi }\log\left\{{\left[\frac{2\alpha^{2}\theta }{\alpha -1}\sum_{k,p,q=0}^{\infty }\delta_{k,p,q}\right]}^{\phi }\cdot \frac{1}{\theta (p+q+1)\phi }\right\},\;\phi >0,\;\phi \neq 1.$$

#### 3.1.6. Tsallis *q*-Entropy

The Tsallis entropy (also called *q*-entropy) of order ϕ for the NAP-HL model, using the expansion in Equation (28), is given by

(40) $$Q_{\phi }=\frac{1}{\phi -1}\left[1-{\left(\frac{2\alpha^{2}\theta }{\alpha -1}\sum_{k,p,q=0}^{\infty }\delta_{k,p,q}\right)}^{\phi }\cdot \frac{1}{\theta (p+q+1)\phi }\right],\;\phi >0,\;\phi \neq 1.$$

Table 2 presents the numerical values of Rényi and Tsallis entropy measures for selected combinations of parameters (α, θ) and entropy orders ϕ in the NAP-HL model.

### 3.2. Order Statistics

Suppose $X_{1},X_{2},\ldots ,X_{n}$ denote the random variables which are independently and identically drawn from the sample sizes *n* with the PDF and CDF defined, respectively, in Equation (17) and Equation (16). The $m^{\mathrm{th}}$-order statistics of those variables $f_{m,k}\left(x\right)$ is defined as

(41) $$f_{m,k}\left(x\right)=\frac{k!}{(m-1)!(k-m)!}f\left(x\right){\left[F\left(x\right)\right]}^{m-1}{\left[1-F\left(x\right)\right]}^{k-m}.$$

Substituting Equations (17) and (12) into Equation (41), one can obtain

(42) $$\begin{array}{cc} f_{m,k}\left(x\right) & =\frac{k!}{(m-1)!(k-m)!}\cdot \frac{2\alpha^{2}\theta }{\alpha -1}\sum_{k,p,q=0}^{\infty }\delta_{k,p,q}\;e^{-\theta (p+q+1)x}\\ \; & \;\times \frac{{\left[\alpha^{1-{\left(\frac{1-e^{-\theta x}}{1+e^{-\theta x}}\right)}^{\alpha }}-\alpha \right]}^{m-1}{\left[1-\alpha^{1-{\left(\frac{1-e^{-\theta x}}{1+e^{-\theta x}}\right)}^{\alpha }}\right]}^{k-m}}{{\left(1-\alpha \right)}^{k-1}} \end{array}$$

which is the $m^{\mathrm{th}}$-order statistics of the NAP-HL model.

## 4. Estimation Approaches

In this section, various frequentist estimation methods are employed to address the problem of estimating the parameters of the NAP-HL model. Parameter estimation serves as a valuable tool for applied statisticians and reliability engineers, offering guidance in selecting an appropriate method for parameter inference. To estimate the parameters of the NAP-HL model, seven estimation techniques were considered, namely, maximum likelihood estimation ($\Delta_{1}$), Cramér–von Mises estimation ($\Delta_{2}$), maximum product of spacings estimation ($\Delta_{3}$), ordinary least squares estimation ($\Delta_{4}$), weighted least squares estimation ($\Delta_{5}$), Anderson–Darling estimation ($\Delta_{6}$), and right-tailed Anderson–Darling estimation ($\Delta_{7}$).

### 4.1. Maximum Likelihood Estimation ($\Delta_{1}$) for Complete Sample

Let the random variables $X_{1},X_{2},\ldots ,X_{n}$ be a random sample from the NAP-HL model with PDF f(x;α,θ), as given in Equation (17). The likelihood function corresponding to f(x;α,θ), say L(α,θ), is given as

(43) $$L\left(x;\alpha ,\theta \right)=\prod_{k=1}^{n}\frac{\alpha \log\alpha }{\alpha -1}{\left(\frac{1-e^{-\theta x}}{1+e^{-\theta x}}\right)}^{\alpha -1}\frac{2\theta e^{-\theta x}}{{\left(1+e^{-\theta x}\right)}^{2}}\;\alpha^{1-{\left(\frac{1-e^{-\theta x}}{1+e^{-\theta x}}\right)}^{\alpha }}$$

Now, the logarithmic likelihood function, say ℓ(x;α,θ), is given as

$$\begin{array}{cc} \ell (x;\alpha ,\theta ) & =n\ln\left(\alpha \right)+n\ln\left(2\theta \right)+n\ln\left(\ln\left(\alpha \right)\right)-n\ln\left(\alpha -1\right)+\left(\alpha -1\right)\sum_{k=1}^{n}\ln\left(1-e^{-\theta x_{k}}\right)-\left(\alpha +1\right)\sum_{k=1}^{n}\ln\left(1+e^{-\theta x_{k}}\right)\\ \; & \;-\theta \sum_{k=1}^{n}x_{k}+\sum_{k=1}^{n}\left[1-{\left(\frac{1-e^{-\theta x_{k}}}{1+e^{-\theta x_{k}}}\right)}^{\alpha }\ln\alpha \right]. \end{array}$$

The derivatives of ℓ(x;α,θ) with respect to α and θ are delineated as

$$\begin{array}{cc} \frac{\partial }{\partial \alpha }\ell \left(x;\alpha ,\gamma ,\eta \right)\; & =\frac{n}{\alpha \ln\alpha }-\frac{n}{\alpha (\alpha -1)}+\sum_{k=1}^{n}\ln\left(1-e^{-\theta x_{k}}\right)-\sum_{k=1}^{n}\ln\left(1-e^{-\theta x_{k}}\right)\\ \; & \;+\sum_{k=1}^{n}\frac{1}{\alpha }\left[1-{\left(\frac{1-e^{-\theta x_{k}}}{1+e^{-\theta x_{k}}}\right)}^{\alpha }\right]-\ln\alpha \sum_{k=1}^{n}\left[{\left(\frac{1-e^{-\theta x_{k}}}{1+e^{-\theta x_{k}}}\right)}^{\alpha }\ln\left(\frac{1-e^{-\theta x_{k}}}{1+e^{-\theta x_{k}}}\right)\right]. \end{array}$$

and

$$\begin{array}{cc} \frac{\partial }{\partial \theta }\ell \left(x;\alpha ,\theta \right)\; & =\frac{n}{2\theta }+\sum_{k=1}^{n}x_{k}+\left(\alpha -1\right)\sum_{k=1}^{n}\frac{x_{k}e^{-\theta x_{k}}}{\ln(1-e^{-\theta x_{k}})}+\left(\alpha +1\right)\sum_{k=1}^{n}\frac{x_{k}e^{-\theta x_{k}}}{\ln(1+e^{-\theta x_{k}})}\\ \; & \;-2\alpha \ln\alpha \sum_{k=1}^{n}{\left(\frac{1-e^{-\theta x_{k}}}{1+e^{-\theta x_{k}}}\right)}^{\alpha -1}\frac{x_{k}e^{-\theta x_{k}}}{{\left(1+e^{-\theta x_{k}}\right)}^{2}}. \end{array}$$

To obtain the maximum likelihood estimates (MLEs) of α and θ, we solve the non-linear system $\frac{\partial }{\partial \alpha }\ell \left(\alpha ,\theta \right)=0$ and $\frac{\partial }{\partial \theta }\ell \left(\alpha ,\theta \right)=0$. These equations are typically solved numerically using iterative methods such as the Newton–Raphson algorithm.

### 4.2. Cramér–Von Mises Estimation ($\Delta_{2}$)

A study conducted in [13] demonstrated that the Cramér–von Mises estimator (CVME) exhibits less bias compared to other minimum distance estimators. In this study, the CVME method is employed to estimate the parameters of the NAP-HL distribution. The CVME of the unknown parameters can be obtained by minimizing the following function:

$$\begin{array}{cc} C(\alpha ,\theta ) & =\frac{1}{12n}+\sum_{k=1}^{n}{\left[F\left(x_{\left(k\right)}\right)-\frac{2k-1}{2n}\right]}^{2},\\ & =\frac{1}{12n}+\sum_{k=1}^{n+1}{\left[\frac{\alpha^{1-{\left(\frac{1-e^{-\theta x_{k}}}{1+e^{-\theta x_{\left(k\right)}}}\right)}^{\alpha }}-\alpha }{1-\alpha }-\frac{2k-1}{2n}\right]}^{2}. \end{array}$$

By evaluating the system of non-linear equations $\frac{\partial }{\partial \alpha }C\left(\alpha ,\theta \right)=0$ and $\frac{\partial }{\partial \theta }C\left(\alpha ,\theta \right)=0$, we obtain the Cramér–von Mises estimates of the parameters of the NAP-HL distribution.

### 4.3. Maximum Product of Spacing Estimation ($\Delta_{3}$)

The maximum-product-of-spacings (MPS) approach was initially introduced in [14] as an alternative to the maximum likelihood estimation method for parameter estimation in continuous univariate distributions. Independently, Reference [15] also explored this method, highlighting its consistency and interpreting it as an estimator of the Kullback–Leibler information measure. By demonstrating the efficiency of the spacing technique and its consistency under broader conditions than those required for maximum likelihood estimation, Reference [14] provided compelling justification for the adoption of the MPS method in this study.

We now proceed to define the uniform spacings for a random sample drawn from the NAP-HL distribution. For a random sample of size *n* with order statistics $X_{\left(1\right)},X_{\left(2\right)},\ldots ,X_{\left(n\right)}$, the uniform spacings are defined as the differences between consecutive order statistics, given by

$$D_{k}=F\left(x_{\left(k\right)}\right)-F\left(x_{\left(k-1\right)}\right)\;;\;k=1,2,...,n+1$$

where $F(x_{\left(0\right)})=0$ and $F(x_{\left(n\right)})=1$. The maximum product of spacings (MPS) estimates for the parameters of the NAP-HL distribution are obtained by maximizing the objective function

$$\begin{array}{cc} M(\alpha ,\theta ) & =\frac{1}{n+1}\sum_{k=1}^{n+1}ln\left[D_{k}\right]\\ & =\frac{1}{n+1}\sum_{k=1}^{n+1}ln\left[\frac{\alpha^{1-{\left(\frac{1-e^{-\theta x_{k}}}{1+e^{-\theta x_{k}}}\right)}^{\alpha }}-\alpha }{1-\alpha }-\frac{\alpha^{1-{\left(\frac{1-e^{-\theta x_{k-1}}}{1+e^{-\theta x_{k-1}}}\right)}^{\alpha }}-\alpha }{1-\alpha }\right]. \end{array}$$

By setting the partial derivatives $\frac{\partial }{\partial \alpha }M\left(\alpha ,\theta \right)=0$ and $\frac{\partial }{\partial \theta }M\left(\alpha ,\theta \right)=0$, we solve the resulting system of non-linear equations to obtain the MPS estimates.

### 4.4. Ordinary Least Square Estimation ($\Delta_{4}$) and Weighted Least Square Estimation ($\Delta_{5}$)

To estimate the parameters of probability models, Reference [16] proposed the use of ordinary least squares and weighted least squares estimation methods. The parameters of the proposed model can be estimated using the least squares estimation approach by minimizing the least squares function S(α,γ,η) with respect to the unknown parameters, where

$$\begin{array}{cc} S(\alpha ,\theta ) & =\sum_{k=1}^{n}{\left[F\left(x_{\left(k\right)}\right)-\frac{k}{n+1}\right]}^{2}\\ & =\sum_{k=1}^{n+1}{\left[\frac{\alpha^{1-{\left(\frac{1-e^{-\theta x_{k}}}{1+e^{-\theta x_{k}}}\right)}^{\alpha }}-\alpha }{1-\alpha }-\frac{k}{n+1}\right]}^{2}. \end{array}$$

Similarly, to obtain the WLS estimate for the unknown parameters of the suggested model, the weighted least square function W(α,θ) is minimized:

$$\begin{array}{cc} W(\alpha ,\theta ) & =\sum_{k=1}^{n}\frac{{\left(n+1\right)}^{2}\left(n+2\right)}{k(n-k+1)}{\left[F\left(x_{\left(k\right)}\right)-\frac{k}{n+1}\right]}^{2}\\ & =\sum_{k=1}^{n}\frac{{\left(n+1\right)}^{2}\left(n+2\right)}{k(n-k+1)}{\left[\frac{\alpha^{1-{\left(\frac{1-e^{-\theta x_{k}}}{1+e^{-\theta x_{k}}}\right)}^{\alpha }}-\alpha }{1-\alpha }-\frac{k}{n+1}\right]}^{2}. \end{array}$$

### 4.5. Anderson–Darling Estimation ($\Delta_{6}$)

The Anderson–Darling test was introduced in [17] as an alternative to traditional statistical methods for assessing whether a sample originates from a non-normal distribution. Later, Reference [18] examined the characteristics of the Anderson–Darling estimator (ADE). Based on his findings, the ADE for the NAP-HL model can be obtained by minimizing the Anderson–Darling statistic, denoted as A(α,θ), which is given by

$$\begin{array}{cc} A(\alpha ,\theta ) & =-n-\frac{1}{n}\sum_{k=1}^{n}\left(2k-1\right)\left[lnF\left(x_{\left(k\right)}\right)+lnS\left(x_{\left(n+1-k\right)}\right)\right]\\ & =-n-\frac{1}{n}\sum_{k=1}^{n}\left(2k-1\right)\left[ln\left(\frac{\alpha^{1-{\left(\frac{1-e^{-\theta x_{k}}}{1+e^{-\theta x_{k}}}\right)}^{\alpha }}-\alpha }{1-\alpha }\right)+ln\left(\frac{1-\alpha^{1-{\left(\frac{1-e^{-\theta x_{n+k-1}}}{1+e^{-\theta x_{n+k-1}}}\right)}^{\alpha }}}{1-\alpha }\right)\right]. \end{array}$$

By evaluating the non-linear equations $\frac{\partial }{\partial \alpha }A\left(\alpha ,\theta \right)=0$ and $\frac{\partial }{\partial \theta }A\left(\alpha ,\theta \right)=0$, we obtain the Anderson–Darling estimates of the parameters of the proposed distribution.

### 4.6. Right-Tailed Anderson–Darling Estimation ($\Delta_{7}$)

The right-tailed Anderson–Darling test, which emphasizes the behavior of the distribution’s right tail, is employed to evaluate the goodness of fit. Using this approach, the parameters α and θ of the NAP-HL distribution are estimated. The right-tailed Anderson–Darling (AD) test statistic, denoted by R(α,θ), is defined as follows:

$$\begin{array}{cc} R(\alpha ,\theta ) & =\frac{n}{2}-2\sum_{k=1}^{n}F\left(x_{\left(k\right)}\right)-\frac{1}{n}\sum_{k=1}^{n}\left(2k-1\right)lnS\left(x_{\left(n+1-k\right)}\right)\\ & =\frac{n}{2}-2\sum_{k=1}^{n}\frac{\alpha^{1-{\left(\frac{1-e^{-\theta x_{k}}}{1+e^{-\theta x_{\left(k\right)}}}\right)}^{\alpha }}-\alpha }{1-\alpha }-\frac{1}{n}\sum_{k=1}^{n}\left(2k-1\right)ln\left(\frac{1-\alpha^{1-{\left(\frac{1-e^{-\theta x_{n+k-1}}}{1+e^{-\theta x_{n+k-1}}}\right)}^{\alpha }}}{1-\alpha }\right). \end{array}$$

## 5. Monte Carlo Simulation for Estimator Evaluation

To rigorously assess the performance of several estimation strategies used for the suggested probability distribution, we conduct a comprehensive Monte Carlo simulation study. The primary aim of this simulation is to compare the estimators in terms of their accuracy, efficiency, and robustness across different sample sizes and scenarios. Each sample was analyzed using the seven estimation methods discussed in the previous section. To compare the performance of these methods, we evaluate the absolute bias (AB), mean square error (MSE) and mean relative error (MRE) for each parameter estimate. The following equations are employed to compute these statistics:

(44) $$\mathrm{AB}=\frac{1}{n}\sum_{i=1}^{n}\left|({\hat{\theta }}_{i}-\theta_{i})\right|\;,$$

(45) $$\mathrm{MSE}=\frac{1}{n}\sum_{i=1}^{n}{\left({\hat{\theta }}_{i}-\theta_{i}\right)}^{2}\;,$$

and

(46) $$\mathrm{MRE}=\frac{1}{n}\sum_{i=1}^{n}|\frac{{\hat{\theta }}_{i}-\theta_{i}}{\theta_{i}}|.$$

To achieve this, we conducted an extensive simulation analysis involving numerous iterations. Random samples of sizes n=25,50,75,100,150,250 and 500 were generated from the proposed model under three distinct parameter settings, as detailed below:

- Set I: α=0.65 and θ=0.95
- Set II: α=1.25 and θ=0.45
- Set III: α=2.15 and θ=1.65.

The outcomes of the Monte Carlo simulation are compiled in Table 3, Table 4 and Table 5. These findings are pivotal for guiding practitioners toward the most reliable estimator for real-world applications of the proposed model.

### Interpretation at the End of the Simulation

A number of inferences can be made from the systematic examination of the simulation findings, such as the following:

- The bias of every estimator, regardless of the estimation technique, decreases as the sample size increases. This suggests that larger samples produce estimates that are more accurate and have less systematic error.
- The mean squared error (MSE) for any method of estimation decreases with a larger sample size. This means increased precision in the estimates resulting from a decrease in variance as well as bias with larger samples.
- Similarly, for all estimators, the mean relative error (MRE) decreases with increasing sample size, demonstrating that larger samples minimize relative errors and yield more accurate and exact estimates.
- Regardless of sample size, the MLE and MPS approaches consistently exhibit the lowest bias, MSE, and MRE, demonstrating their superior dependability for parameter estimation. These techniques have low errors across sample sizes and are very effective.
- In comparison to MLE and MPS, the CVM approach consistently has higher bias, MSE, and MRE, albeit it shows some improvement with bigger sample numbers. Therefore, CVM is not as accurate or efficient as MLE or MPS, even if it might become better with larger samples.
- Particularly for smaller sample sizes, the OLS and WLS approaches typically have the highest bias, MSE, and MRE. This indicates that these techniques may not be as appropriate for high-quality estimation in any situation because of their lower accuracy and precision when compared to MLE and MPS.

## 6. Data Fitting

In this section, Two real datasets are evaluated to prove the flexibility of the NAP-HL distribution. Test statistics for the NAP-HL distribution and other competitive distributions are calculated. The NAP-HL distribution is compared with other distributions, namely, the odd Frechet half-logistic (OFHL) distribution [19], the Kumaraswamy half-logistic (KHL) distribution [20], the exponentiated half-logistic (EHL) distribution [21], the Marshall–Olkin half-logistic (MoHL) distribution [22], the half-logistic (HL) distribution [23], and the power half-logistic (PoHL) distribution [24].

For clarity and completeness, the analytical expressions of the probability density functions (PDFs) of the competing models are presented below:

- The odd Frechet half-logistic (OFHL) distribution’s PDF is given as
  (47) $$f\left(x;\alpha ,\gamma \right)=\frac{\alpha \gamma {\left(2e^{-\gamma x}\right)}^{\alpha }}{{\left(1-e^{-\gamma x}\right)}^{\alpha +1}}\exp\left\{-{\left(\frac{2e^{-\gamma x}}{1-e^{-\gamma x}}\right)}^{\alpha }\right\}$$
- The Kumaraswamy half-logistic (KHL) distribution’s PDF is given as
  (48) $$f\left(x;\alpha ,\beta ,\gamma \right)=\frac{2\alpha \beta \gamma e^{-\gamma x}}{{\left(1+e^{-\gamma x}\right)}^{2}}{\left(\frac{1-e^{-\gamma x}}{1+e^{-\gamma x}}\right)}^{\alpha -1}{\left[1-{\left(\frac{1-e^{-\gamma x}}{1+e^{-\gamma x}}\right)}^{\alpha }\right]}^{\beta -1}$$
- The exponentiated half-logistic (EHL) distribution’s PDF is given as
  (49) $$f\left(x;\alpha ,\gamma \right)=\frac{2\alpha \gamma e^{-\gamma x}}{1-e^{-2\gamma x}}{\left(\frac{1-e^{-\gamma x}}{1+e^{-\gamma x}}\right)}^{\alpha }$$
- The Marshall–Olkin half-logistic (MoHL) distribution’s PDF is given as
  (50) $$f\left(x;\alpha ,\gamma \right)=\frac{2\alpha \gamma e^{-\gamma x}}{{\left[1+\left(2\alpha -1\right)e^{-\gamma x}\right]}^{2}}$$
- The half-logistic (HL) distribution’s PDF is given as
  (51) $$f\left(x;\gamma \right)=\frac{2\gamma e^{-\gamma x}}{{\left(1+e^{-\gamma x}\right)}^{2}}$$
- The power half-logistic (PoHL) distribution’s PDF is given as
  (52) $$f\left(x;\alpha ,\gamma \right)=\frac{2\alpha \gamma x^{\alpha -1}e^{-\gamma x^{\alpha }}}{{\left[1+e^{-\gamma x^{\alpha }}\right]}^{2}}$$

### 6.1. Application in Metrology

A set of 20 component failure times, known as the SDS dataset, was analyzed in [25] using the Pareto distribution and Bayesian prediction techniques. The observed failure times are as follows: 0.0009, 0.0040, 0.0142, 0.0221, 0.0261, 0.0418, 0.0473, 0.0834, 0.1091, 0.1252, 0.1404, 0.1498, 0.1750, 0.2031, 0.2099, 0.2168, 0.2918, 0.3465, 0.4035, and 0.6143. These values represent the time-to-failure measurements collected in ascending order and exhibited right-skewed behavior, which may suggest increasing or bathtub-shaped hazard rates. The authors modeled the dataset using the Pareto distribution and employed a Bayesian prediction function to make inferences about component reliability and future failure behavior.

### 6.2. Application in Engineering

The data represent the fatigue fracture life of Kevlar 373/epoxy subjected to constant pressure at the 90-stress level until all specimens failed. This dataset includes seventy-six observations and was analyzed in [26]. The observations are as follows: 0.0251, 0.0886, 0.0891, 0.2501, 0.3113, 0.3451, 0.4763, 0.5650, 0.5671, 0.6566, 0.6748, 0.6751, 0.6753, 0.7696, 0.8375, 0.8391, 0.8425, 0.8645, 0.8851, 0.9113, 0.9120, 0.9836, 1.0483, 1.0596, 1.0773, 1.1733, 1.2570, 1.2766, 1.2985, 1.3211, 1.3503, 1.3551, 1.4595, 1.4880, 1.5728, 1.5733, 1.7083, 1.7263, 1.7460, 1.7630, 1.7746, 1.8275, 1.8375, 1.8503, 1.8808, 1.8878, 1.8881, 1.9316, 1.9558, 2.0048, 2.0408, 2.0903, 2.1093, 2.1330, 2.2100, 2.2460, 2.2878, 2.3203, 2.3470, 2.3513, 2.4951, 2.5260, 2.9911, 3.0256, 3.2678, 3.4045, 3.4846, 3.7433, 3.7455, 3.9143, 4.8073, 5.4005, 5.4435, 5.5295, 6.5541, 9.0960.

The descriptive properties of the metrology and engineering data are summarized in Table 6 and Table 7. Table 6 summarizes the metrology dataset, which contains 20 observations with values ranging from 0.0009 to 0.6143. The mean and standard deviation are 0.16126 and 0.15733, respectively, indicating moderate dispersion. Table 7, on the other hand, details the engineering dataset of 76 observations, showing a much wider range (0.0251 to 9.0960) and higher average (mean = 1.9592, SD = 1.5740), reflecting greater variability.

### 6.3. Evaluation Criterion

This section presents the application of the NAP-HL distribution to two real-life datasets. The unknown parameters of the NAP-HL distribution are estimated using the maximum likelihood estimation (MLE) method. To assess the goodness of fit and compare the performance of the model, several evaluation criteria are employed. These include the maximized log-likelihood value ($\hat{\ell }$), Anderson–Darling statistic ($A^{*}$), Cramér–von Mises statistic ($W^{*}$) and Kolmogorov–Smirnov statistic (K-S) along with its associated *p*-value. Additionally, information criteria such as the Akaike information criterion (AIC) and Bayesian information criterion (BIC) are used for model comparison. All computations are performed using the R software version 4.3.2 and the results are reported in Table 8 and Table 9. Furthermore, we fitted the NAP-HL distribution by utilizing seven estimation procedures and the results are reported in Table 10 and Table 11.

**Figure 3:**
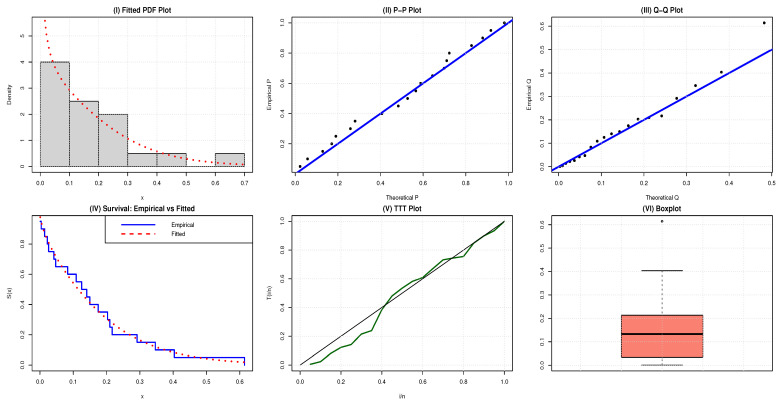
Comparative plots for evaluating the adequacy of the NAP-HL model using metrology data.

**Figure 4:**
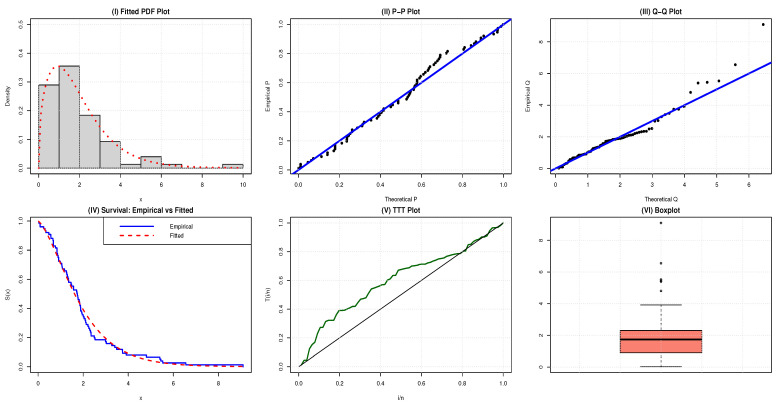
Comparative plots for evaluating the adequacy of the NAP-HL model using engineering data.

The estimated PDF, P–P plots, Q–Q plots, survival function (SF), TTT plots and box plots of the NAP-HL model for the two datasets are contrasted in Figure 3 and Figure 4, respectively.

### 6.4. Results and Discussion

Box plots are a standard way to represent the summary statistics of a data distribution. In Figure 3 and Figure 4, the box plots for dataset 1 and dataset 2 are presented. Both datasets are right-skewed with some outliers, highlighting the extreme behavior in the data distributions.

Table 8 and Table 9 report the values of the maximized log-likelihood ($\hat{\ell }$), AIC, BIC, Anderson–Darling ($A^{*}$), Cramér–von Mises ($W^{*}$), and Kolmogorov–Smirnov (K-S) statistics and the corresponding *p*-values for both the datasets, respectively.

The distribution with the smallest values of all the information criteria and the largest *p*-value is typically considered the best fit. Based on these criteria, the superiority of the NAP-HL model over the competing models is evident.

For a more comprehensive graphical representation, several parametric and non-parametric plots are provided in Figure 3 and Figure 4. These include the fitted PDF, probability–probability (P–P) plots, quantile–quantile (Q–Q) plots, survival function (SF), total time on test (TTT) plots, and box plots of the NAP-HL distribution for each dataset. The figures demonstrate that the NAP-HL distribution fits both the datasets very well.

The failure rate of application 1 is bathtub-shaped because the TTT-transform plot displayed in Figure 3 is initially convex below the 45° line and then concave above the 45° line and the box plot has few outliers, as shown in Figure 3.

The TTT-transform plot of application 2 shows a concave pattern above the 45° line, providing insight into the shape of the hazard function, as shown in Figure 2 and reveals the presence of outliers in the box plot illustrated in Figure 4.

## 7. Concluding Remarks

Continuous data in many disciplines demand more flexible lifetime models than those offered by the classical half-logistic law. We introduce the NAP-HL (novel alpha-power half-logistic) distribution, a three-parameter extension that embeds a shape control into the standard half-logistic form. By adjusting this shape parameter, the NAP-HL model can produce increasing, decreasing, or bathtub-shaped hazard rates, thus capturing a wide range of real-world aging and failure behaviors.

We derive closed-form expressions for the main characteristics of the NAP-HL model—moments, quantile function, and hazard function—highlighting how the added flexibility emerges from the exponentiation mechanism. A comprehensive Monte Carlo study compares seven estimation methods revealing their relative performance under different sample sizes and true parameter settings.

To assess empirical usefulness, we fit the NAP-HL model to two benchmark datasets: a metrology and an engineering dataset. In every case, the NAP-HL model achieves lower AIC and BIC and superior goodness-of-fit statistics (AD, CvM, K-S) than its half-logistic precursor and several competing families.

Future work will extend the NAP-HL model into regression frameworks, accommodate censored and truncated data streams, explore Bayesian estimation for small samples, and integrate with machine learning pipelines for large-scale predictive analytics. Such enhancements promise to make the NAP-HL model a go-to model for complex reliability and survival analysis tasks.

## Figures and Tables

**Table 1:** Summary of moments, mode, and shape measures for selected (α,θ) parameter combinations.

α	θ	$\mu_{1}$	$\mu_{2}$	$\mu_{3}$	$\mu_{4}$	Mode	Variance	Skewness	Kurtosis
1.1	0.6	2.3934	9.5453	52.4483	367.6041	0.6715	3.8168	1.5197	6.5289
	1.4	1.0258	1.7532	4.1286	12.4015	0.2878	0.7010		
	2.0	0.7180	0.8591	1.4161	2.9776	0.2014	0.3435		
	2.8	0.5129	0.4383	0.5161	0.7751	0.1439	0.1753		
1.8	0.6	2.8543	11.9338	66.3812	466.0194	1.6595	3.7868	1.4523	6.4409
	1.4	1.2233	2.1919	5.2253	15.7216	0.7112	0.6955		
	2.0	0.8563	1.0740	1.7923	3.7748	0.4979	0.3408		
	2.8	0.6116	0.5480	0.6532	0.9826	0.3556	0.1739		
2.6	0.6	3.2316	14.0938	79.1893	555.0914	2.1977	3.6508	1.4406	6.5567
	1.4	1.3850	2.5887	6.2336	18.7265	0.9419	0.6706		
	2.0	0.9695	1.2684	2.1381	4.4962	0.6593	0.3286		
	2.8	0.6925	0.6472	0.7792	1.1704	0.4709	0.1676		

**Table 2:** Numerical evaluation of Rényi and Tsallis entropy measures of order ϕ=1.5, 2.0, and 2.75 for various combinations of α and θ parameters in the NAP-HL model.

α	θ	ϕ=1.50		ϕ=2.0		ϕ=2.75	
		Rényi	Tsallis	Rényi	Tsallis	Rényi	Tsallis
1.10	0.60	1.7172	1.1524	1.6441	0.8068	1.5760	0.5351
	1.40	0.8699	0.7054	0.7968	0.5492	0.7287	0.4118
	2.00	0.5132	0.4526	0.4401	0.3560	0.3721	0.2735
	2.80	0.1767	0.1691	0.1036	0.0984	0.0356	0.0345
1.80	0.60	1.7909	1.1832	1.7201	0.8209	1.6531	0.5398
	1.40	0.9436	0.7523	0.8728	0.5822	0.8058	0.4319
	2.00	0.5870	0.5087	0.5161	0.4032	0.4491	0.3110
	2.80	0.2504	0.2354	0.1796	0.1644	0.1127	0.1023
2.60	0.60	1.7917	1.1835	1.7173	0.8205	1.6467	0.5394
	1.40	0.9444	0.7528	0.8701	0.5811	0.7995	0.4304
	2.00	0.5877	0.5093	0.5133	0.4015	0.4428	0.3081
	2.80	0.2512	0.2361	0.1769	0.1621	0.1063	0.0970

**Table 3:** Performance metrics of estimators via simulation for α=0.65 and θ=0.95.

Metric	Param	Sample Size	$\Delta_{1}$	$\Delta_{2}$	$\Delta_{3}$	$\Delta_{4}$	$\Delta_{5}$	$\Delta_{6}$	$\Delta_{7}$
AB	α	25	0.16952	0.22655	0.15789	0.22459	0.22850	0.19030	0.33279
		50	0.11812	0.13224	0.12185	0.15757	0.14094	0.13824	0.19588
		75	0.10194	0.12100	0.09740	0.11880	0.11689	0.10164	0.12982
		100	0.08381	0.09920	0.07882	0.10297	0.08637	0.08545	0.11325
		150	0.06724	0.08571	0.07030	0.08591	0.06689	0.07203	0.08639
		250	0.04904	0.06491	0.05287	0.06910	0.05003	0.05606	0.07554
		500	0.03472	0.03802	0.03530	0.04128	0.04319	0.03921	0.04796
	θ	25	0.15560	0.19776	0.18027	0.20890	0.17780	0.15132	0.19716
		50	0.11296	0.12976	0.12421	0.12789	0.13570	0.11746	0.12021
		75	0.09862	0.11429	0.08742	0.11170	0.10299	0.09446	0.11351
		100	0.08416	0.09726	0.07233	0.09335	0.08712	0.08765	0.08310
		150	0.06793	0.07787	0.06687	0.07482	0.06387	0.06935	0.07755
		250	0.04402	0.05980	0.05090	0.05815	0.04708	0.04433	0.05465
		500	0.03609	0.04238	0.03127	0.03858	0.03995	0.03847	0.03645
MSE	α	25	0.05771	0.11947	0.04205	0.10684	0.11344	0.08579	0.32714
		50	0.02488	0.03288	0.02821	0.04861	0.03468	0.02708	0.07733
		75	0.01803	0.03060	0.01414	0.02739	0.02296	0.01716	0.03764
		100	0.01107	0.01559	0.01051	0.01807	0.01301	0.01208	0.02034
		150	0.00742	0.01173	0.00739	0.01249	0.00744	0.00797	0.01095
		250	0.00387	0.00643	0.00443	0.00790	0.00422	0.00475	0.00912
		500	0.00193	0.00249	0.00182	0.00266	0.00288	0.00223	0.00366
	θ	25	0.04472	0.07113	0.04988	0.08305	0.04913	0.03985	0.06601
		50	0.02117	0.02517	0.02092	0.02725	0.02936	0.02256	0.02502
		75	0.01631	0.02208	0.01114	0.01966	0.01701	0.01524	0.02137
		100	0.01118	0.01517	0.00823	0.01355	0.01390	0.01196	0.01182
		150	0.00704	0.00960	0.00638	0.00882	0.00690	0.00819	0.00980
		250	0.00313	0.00506	0.00407	0.00537	0.00363	0.00313	0.00437
		500	0.00199	0.00288	0.00163	0.00259	0.00243	0.00234	0.00207
MRE	α	25	0.26081	0.34853	0.24291	0.34552	0.35153	0.29277	0.51199
		50	0.18172	0.20345	0.18746	0.24242	0.21683	0.21268	0.30135
		75	0.15683	0.18616	0.14984	0.18277	0.17983	0.15638	0.19972
		100	0.12894	0.15262	0.12126	0.15841	0.13287	0.13146	0.17422
		150	0.10345	0.13186	0.10815	0.13217	0.10290	0.11081	0.13290
		250	0.07545	0.09986	0.08133	0.10631	0.07697	0.08624	0.11621
		500	0.05342	0.05849	0.05431	0.06350	0.06644	0.06032	0.07379
	θ	25	0.16379	0.20817	0.18975	0.21989	0.18716	0.15928	0.20754
		50	0.11891	0.13659	0.13075	0.13462	0.14284	0.12364	0.12653
		75	0.10381	0.12031	0.09202	0.11758	0.10842	0.09943	0.11949
		100	0.08859	0.10238	0.07614	0.09826	0.09171	0.09226	0.08747
		150	0.07150	0.08197	0.07039	0.07876	0.06723	0.07300	0.08163
		250	0.04633	0.06295	0.05358	0.06121	0.04956	0.04667	0.05752
		500	0.03799	0.04461	0.03292	0.04061	0.04205	0.04050	0.03837

**Table 4:** Performance metrics of estimators via simulation for α=1.25 and θ=0.45.

Metric	Param	Sample Size	$\Delta_{1}$	$\Delta_{2}$	$\Delta_{3}$	$\Delta_{4}$	$\Delta_{5}$	$\Delta_{6}$	$\Delta_{7}$
AB	α	25	0.31304	0.41252	0.28567	0.40247	0.41101	0.33372	0.60000
		50	0.21002	0.23263	0.22311	0.28188	0.26343	0.23891	0.35346
		75	0.18287	0.22633	0.17074	0.21304	0.20236	0.18030	0.23498
		100	0.15301	0.17823	0.14055	0.18270	0.15748	0.15785	0.19867
		150	0.12343	0.14998	0.12678	0.15616	0.11922	0.12420	0.16371
		250	0.08678	0.11651	0.09072	0.12614	0.08514	0.09605	0.13188
		500	0.06242	0.07059	0.06185	0.07326	0.07878	0.07247	0.08558
	θ	25	0.06463	0.07860	0.07335	0.08155	0.07107	0.06223	0.08062
		50	0.04658	0.05280	0.04967	0.05008	0.05407	0.04766	0.04945
		75	0.03971	0.04555	0.03489	0.04464	0.04185	0.03829	0.04758
		100	0.03479	0.03873	0.02840	0.03754	0.03472	0.03490	0.03470
		150	0.02715	0.03156	0.02730	0.02978	0.02596	0.02817	0.03210
		250	0.01809	0.02408	0.02095	0.02318	0.01946	0.01834	0.02307
		500	0.01443	0.01705	0.01261	0.01546	0.01590	0.01540	0.01540
MSE	α	25	0.19597	0.37436	0.13941	0.33277	0.38629	0.26307	1.07065
		50	0.08130	0.10202	0.09123	0.15054	0.11877	0.08623	0.25440
		75	0.06089	0.10026	0.04509	0.08452	0.06649	0.05562	0.12613
		100	0.03686	0.05070	0.03437	0.05799	0.04284	0.03946	0.06244
		150	0.02481	0.03816	0.02434	0.04023	0.02366	0.02386	0.03800
		250	0.01218	0.02001	0.01375	0.02582	0.01291	0.01406	0.02808
		500	0.00628	0.00862	0.00569	0.00845	0.00950	0.00750	0.01145
	θ	25	0.00773	0.01041	0.00811	0.01214	0.00780	0.00645	0.01054
		50	0.00353	0.00412	0.00333	0.00414	0.00457	0.00371	0.00426
		75	0.00266	0.00351	0.00178	0.00312	0.00283	0.00247	0.00371
		100	0.00190	0.00238	0.00130	0.00219	0.00224	0.00191	0.00205
		150	0.00113	0.00152	0.00106	0.00138	0.00114	0.00133	0.00170
		250	0.00051	0.00082	0.00068	0.00084	0.00062	0.00052	0.00076
		500	0.00032	0.00046	0.00027	0.00042	0.00039	0.00038	0.00036
MRE	α	25	0.25043	0.33002	0.22854	0.32197	0.32881	0.26698	0.48000
		50	0.16802	0.18610	0.17849	0.22551	0.21074	0.19113	0.28277
		75	0.14629	0.18106	0.13659	0.17043	0.16189	0.14424	0.18798
		100	0.12240	0.14258	0.11244	0.14616	0.12598	0.12628	0.15894
		150	0.09874	0.11998	0.10142	0.12493	0.09538	0.09936	0.13097
		250	0.06943	0.09321	0.07258	0.10091	0.06811	0.07684	0.10551
		500	0.04994	0.05647	0.04948	0.05861	0.06303	0.05798	0.06847
	θ	25	0.14362	0.17466	0.16299	0.18123	0.15792	0.13829	0.17916
		50	0.10352	0.11734	0.11038	0.11130	0.12015	0.10591	0.10990
		75	0.08825	0.10121	0.07754	0.09921	0.09301	0.08510	0.10573
		100	0.07732	0.08606	0.06311	0.08342	0.07715	0.07755	0.07712
		150	0.06032	0.07014	0.06067	0.06619	0.05769	0.06260	0.07134
		250	0.04019	0.05351	0.04655	0.05152	0.04324	0.04075	0.05127
		500	0.03206	0.03790	0.02803	0.03436	0.03534	0.03422	0.03422

**Table 5:** Performance metrics of estimators via simulation for α=2.15 and θ=1.65.

Metric	Param	Sample Size	$\Delta_{1}$	$\Delta_{2}$	$\Delta_{3}$	$\Delta_{4}$	$\Delta_{5}$	$\Delta_{6}$	$\Delta_{7}$
AB	α	25	0.55055	0.71109	0.49159	0.69749	0.69949	0.55309	1.00703
		50	0.35852	0.39782	0.38282	0.48111	0.46451	0.39221	0.59128
		75	0.30933	0.39155	0.28167	0.36096	0.33510	0.30122	0.40971
		100	0.26742	0.30689	0.23537	0.30509	0.27776	0.27438	0.32518
		150	0.21342	0.24973	0.21635	0.26569	0.20155	0.20752	0.28648
		250	0.14413	0.19844	0.14756	0.21450	0.14414	0.15747	0.21723
		500	0.10599	0.12460	0.10112	0.12196	0.13471	0.12667	0.14217
	θ	25	0.21377	0.25540	0.24153	0.26232	0.22994	0.20630	0.26704
		50	0.15487	0.17314	0.15945	0.16194	0.17598	0.15647	0.16562
		75	0.12914	0.14830	0.11235	0.14575	0.13751	0.12516	0.15975
		100	0.11464	0.12604	0.09068	0.12228	0.11316	0.11316	0.11624
		150	0.08764	0.10313	0.08932	0.09684	0.08531	0.09207	0.10694
		250	0.05974	0.07856	0.06823	0.07538	0.06412	0.06073	0.07764
		500	0.04665	0.05567	0.04194	0.05040	0.05166	0.05029	0.05174
MSE	α	25	0.61585	1.10949	0.41627	0.98083	1.15792	0.74203	2.98027
		50	0.24043	0.29046	0.26232	0.42290	0.36655	0.24993	0.73395
		75	0.18509	0.29542	0.12586	0.24206	0.17928	0.16207	0.37413
		100	0.11085	0.14989	0.09838	0.16601	0.12973	0.11575	0.17376
		150	0.07401	0.11185	0.07068	0.11523	0.06835	0.06693	0.11737
		250	0.03419	0.05638	0.03845	0.07409	0.03613	0.03777	0.07584
		500	0.01821	0.02674	0.01585	0.02444	0.02784	0.02254	0.03180
	θ	25	0.08502	0.10635	0.08649	0.12237	0.08218	0.06890	0.11315
		50	0.03815	0.04403	0.03489	0.04301	0.04789	0.03969	0.04761
		75	0.02823	0.03700	0.01876	0.03299	0.03055	0.02637	0.04156
		100	0.02063	0.02494	0.01348	0.02318	0.02379	0.02024	0.02295
		150	0.01195	0.01603	0.01156	0.01457	0.01229	0.01404	0.01898
		250	0.00553	0.00871	0.00733	0.00888	0.00668	0.00567	0.00856
		500	0.00332	0.00487	0.00285	0.00443	0.00411	0.00408	0.00409
MRE	α	25	0.25607	0.33074	0.22865	0.32441	0.32534	0.25725	0.46839
		50	0.16676	0.18503	0.17806	0.22377	0.21605	0.18242	0.27501
		75	0.14388	0.18212	0.13101	0.16789	0.15586	0.14010	0.19056
		100	0.12438	0.14274	0.10948	0.14190	0.12919	0.12762	0.15125
		150	0.09927	0.11615	0.10063	0.12357	0.09375	0.09652	0.13325
		250	0.06704	0.09230	0.06863	0.09977	0.06704	0.07324	0.10104
		500	0.04930	0.05795	0.04703	0.05672	0.06266	0.05892	0.06613
	θ	25	0.12956	0.15479	0.14638	0.15898	0.13936	0.12503	0.16184
		50	0.09386	0.10493	0.09664	0.09814	0.10665	0.09483	0.10037
		75	0.07827	0.08988	0.06809	0.08833	0.08334	0.07585	0.09682
		100	0.06948	0.07639	0.05496	0.07411	0.06858	0.06858	0.07045
		150	0.05312	0.06251	0.05413	0.05869	0.05171	0.05580	0.06481
		250	0.03621	0.04761	0.04135	0.04569	0.03886	0.03681	0.04705
		500	0.02827	0.03374	0.02542	0.03054	0.03131	0.03048	0.03135

**Table 6:** Descriptive statistics of the metrology data.

Count	Smallest	Largest	Mean	Median	Std. Dev.	Variance	Kurtosis	Skewness	Range
20	0.0009	0.6143	0.16126	0.1328	0.15733	0.02475	2.34733	1.44060	0.6134

**Table 7:** Descriptive statistics of the engineering data.

Count	Smallest	Largest	Mean	Median	Std. Dev.	Variance	Kurtosis	Skewness	Range
76	0.0251	9.0960	1.9592	1.7362	1.5740	2.4774	5.6004	2.0196	9.0709

**Table 8:** Parameter estimates and goodness-of-fit metrics for competing models applied to metrology data.

Model	α	γ	β	−ℓ	AIC	BIC	$A^{*}$	$W^{*}$	K-S	*p*-Value
NAP-HL	0.6169	7.1682	–	17.7517	−31.5033	−29.5119	0.1263	0.0209	0.0835	0.9969
OFHL	0.3083	21.1333	–	17.2153	−30.3970	−28.4056	0.2966	0.0588	0.1335	0.8227
KHL	0.6469	8.5419	0.7706	17.2076	−28.4306	−25.4434	0.1369	0.0236	0.0909	0.9911
EHL	0.6774	6.7704	–	16.4971	−30.4152	−28.4237	0.1431	0.0248	0.0943	0.9867
MoHL	0.4734	6.0389	–	16.0246	−28.9942	−27.0027	0.3344	0.0424	0.0972	0.9818
HL	–	8.3243	–	16.9198	−30.0491	−29.0534	0.6561	0.0675	0.1556	0.6618
PoHL	0.7841	6.2442	–	17.2517	−29.8396	−27.8481	0.2032	0.0327	0.1002	0.9758

**Table 9:** Parameter estimates and goodness-of-fit metrics for competing models applied to engineering data.

Model	α	γ	β	−ℓ	AIC	BIC	$A^{*}$	$W^{*}$	K-S	*p*-Value
NAP-HL	1.4020	0.8065	–	121.692	247.384	252.045	0.5350	0.0896	0.0922	0.5083
OFHL	0.5154	1.1917	–	127.906	259.812	264.473	2.5326	0.4881	0.1543	0.0479
KHL	1.3834	0.5019	1.4250	121.316	248.632	255.624	0.5622	0.0906	0.0977	0.4352
EHL	1.3308	0.8399	–	121.776	247.552	252.213	0.5859	0.0944	0.1000	0.4068
MoHL	1.7273	0.9111	–	122.421	248.842	253.504	0.7670	0.1224	0.1043	0.3555
HL	0.7278	–	–	123.403	248.807	251.138	1.4097	0.2522	0.1161	0.2383
PoHL	1.1271	0.6353	–	122.565	249.131	253.792	0.8190	0.1366	0.1076	0.3197

**Table 10:** Comparative analysis of estimation methods and associated GoF metrics for the NAP-HL model using metrology data.

Method	α	γ	−ℓ	AIC	BIC	$A^{*}$	$W^{*}$	K-S	*p*-Value
MLE	0.6168	7.1685	17.7517	−31.5033	−29.5119	0.1263	0.0209	0.0835	0.9969
CVME	0.5954	6.8486	17.2332	−30.4664	−28.4750	0.1266	0.0208	0.0893	0.9928
MPSE	0.4728	5.9935	16.8914	−29.7827	−27.7912	0.1258	0.0205	0.1031	0.9687
OLSE	0.5207	6.2630	17.0673	−30.1347	−28.1432	0.1263	0.0207	0.1021	0.9714
WLSE	0.5020	6.1909	17.0164	−30.0327	−28.0413	0.1259	0.0206	0.1008	0.9744
ADE	0.5848	6.8648	17.2318	−30.4637	−28.4722	0.1260	0.0206	0.0860	0.9954
RTADE	0.6309	7.1105	17.2473	−30.4946	−28.5031	0.1270	0.0209	0.0834	0.9969

**Table 11:** Comparative analysis of estimation methods and associated GoF metrics for the NAP-HL model using engineering data.

Method	α	γ	−ℓ	AIC	BIC	$A^{*}$	$W^{*}$	K-S	*p*-Value
MLE	1.4022	0.8065	121.6920	247.3839	252.0454	0.5350	0.0896	0.0922	0.5083
CVME	1.8027	0.9181	123.0494	250.0989	254.7604	0.5615	0.0949	0.0674	0.8566
MPSE	1.2513	0.7598	121.9393	247.8787	252.5402	0.6531	0.1105	0.1096	0.2988
OLSE	1.7361	0.9024	122.6746	249.3491	254.0106	0.5692	0.0962	0.0712	0.8091
WLSE	1.6373	0.8783	122.2174	248.4348	253.0963	0.5819	0.0983	0.0770	0.7288
ADE	1.5725	0.8582	121.9720	247.9440	252.6055	0.5918	0.1000	0.0831	0.6389
RTADE	1.5485	0.8550	121.9165	247.8329	252.4944	0.5950	0.1005	0.0828	0.6442

## Data Availability

The data supporting the findings of this study are available within the article.
